# Performance Evaluation of Pebble Concrete for Pavement: A Study on the Sucre Highway Project

**DOI:** 10.3390/ma17204994

**Published:** 2024-10-12

**Authors:** Zhuqing Feng, Jue Lu, Simin Liu, Jingliang Xia, Jing Wang, Faguang Leng, Xinxin Ma

**Affiliations:** 1 China Harbour Engineering Co., Ltd., Beijing 100027, China; 2 Institute of Building Materials, China Academy of Building Research, Beijing 100013, China

**Keywords:** pavement concrete, pebble aggregate, fly ash, workability, mechanical performance, impermeability

## Abstract

Bolivia has abundant pebbles, while the supply of crushed stone is limited and unstable. Thus, the resource utilization of local pebble as a coarse aggregate and the guarantee of concrete durability are the key scientific issues in the Sucre Highway Project. In this paper, a comparative analysis was conducted of the performance of crushed stone concrete and pebble concrete. Additionally, the impact of fly ash on the water permeability resistance of concrete was investigated. The results indicate that the apparent density, bulk density, and void ratio of pebbles are lower than those of crushed stone, and the aggregate gradation of pebbles is dispersed. The type of aggregate is the primary factor influencing the splitting tensile strength of concrete, with the main failure modes of pebble concrete being slurry cracking, aggregate crushing, and interface debonding. While aggregate and fly ash have a minor effect on compressive strength, they significantly impact flexural tensile strength; however, all concretes meet the requirements for extra-heavy, very heavy, and heavy traffic load levels. In terms of impermeability, fly ash effectively mitigates the negative impact of aggregate type on the impermeability of concrete. These findings support the application of pebble concrete in the highway project.

## 1. Introduction

As global infrastructure development advances, there is an increasing emphasis on utilizing local resources in road construction to reduce costs and promote sustainability [1]. However, due to variations in geography, climate, and material properties, the selection and application of construction materials can differ significantly across regions. The Sucre Highway Project in Bolivia, a crucial segment of National Highway 6, exemplifies this approach. Due to frequent traffic congestion on the existing two-lane road, the Administradora Boliviana de Carreteras (ABC) has planned the widening and upgrading of the highway, making the selection of coarse aggregate a critical aspect of material procurement.

As a crucial component of concrete, aggregates primarily serve as a structural filler, constituting 60 vol% to 80 vol% of the concrete [2]. Given the varying distribution of resources across regions, studying the performance of pebble-based concrete is essential for the successful execution of construction projects [3,4]. Pebbles, when used as coarse aggregate, exhibit distinct physical characteristics compared to conventional crushed stone, leading to notable differences in their application in concrete. While changes in the shape characteristics of coarse aggregates typically have minimal effects on the workability of fresh concrete, the mineral composition of the aggregate and its surface texture significantly influence the microstructure of the interfacial transition zone (ITZ) between the paste and the aggregate, subsequently impacting the mechanical properties of the concrete. Previous studies have shown that pebble-based concrete tends to exhibit inferior tensile and compressive strengths compared to concrete made with crushed stone [5,6,7]. The quantitative analysis of aggregate morphological characteristics provides a more effective method for predicting the influence of coarse aggregate on the rheological properties and mechanical properties of concrete [8]. However, the composition and surface characteristics of the aggregate parent rock will affect the microstructure of the transition zone, which will affect the mechanical properties of concrete [9,10]. Research [11,12] has shown that the mechanical properties of pebble concrete are lower than those of crushed stone concrete, particularly its tensile and compressive properties. According to He et al. [13], the fractal dimension of the concrete interface has a good correlation with the mechanical properties of new and old concrete, such as the splitting tensile strength, bending strength, and bonding strength. Li et al. [14] tested the interface bond strength and found that with the increase in aggregate surface roughness, the tensile and shear bond strength of the interface increased and tended to be constant. In addition, the maximum particle size and grading of pebbles also have a significant impact on the strength of concrete [15].

Although studies on the long-term and durability performance of pebble-based concrete have primarily focused on volume stability [16,17], there is limited research addressing the permeability properties that directly influence its long-term service performance. In fact, pebble concrete, due to its lower impermeability, may negatively impact the long-term service life of roads when exposed to harsh environments. Previous studies [18,19] have shown that fly ash can improve the pore structure of concrete through its filling effect and pozzolanic reaction, significantly enhancing its impermeability and durability.

This study investigates the influence of aggregate properties on the mechanical performance of concrete by conducting a comparative analysis of two local pebbles in Bolivia and domestic crushed stone. At the same time, in order to ensure the durability of concrete, the preparation of concrete with fly ash as a mineral admixture is considered. Specifically, the study evaluates the splitting tensile strength, compressive strength, and flexural tensile strength of concrete made with these aggregates, along with their permeability properties. The findings aim to provide valuable technical support for optimizing the use of local pebble resources in the Sucre Highway Project, promoting both efficient resource utilization and enhanced concrete performance.

## 2. Project Overview

### 2.1. Project Description

**Figure 1:**
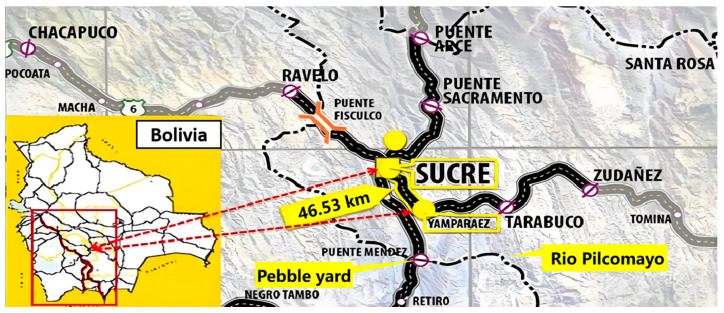
Project location diagram.

Figure 1 illustrates the location of the Sucre Highway Project in Bolivia, which spans a total length of 25.84 km. The route begins at the end of Avenida del Ejército Nacional in Sucre, passing through the town of Yampares and terminating at the first intersection with a village road. The project is marked by stationing points from KM 345 + 320 to KM 371 + 176.06. The work’s scope includes widening and upgrading the existing road, comprising subgrade construction, pavement works, bridge and culvert construction, traffic safety systems, and environmental impact assessments. The total length of the concrete pavement is 26,000 linear meters, requiring approximately 107,000 m^3^ of concrete.

### 2.2. Geographic and Climatic Conditions

Sucre, the project location, is situated in south–central Bolivia, approximately 40 km from the Rio Pilcomayo, one of Bolivia’s major rivers. The spring and summer seasons span from September to February, with temperatures ranging from 7 °C to 32 °C. The autumn and winter seasons, from March to August, experience cooler temperatures ranging from 1 °C to 15 °C. The climate of the project is suitable all year round.

### 2.3. Main Local Materials

The main materials for pavement concrete include cement, fine aggregates, and coarse aggregates. These bulk materials are purchased locally. Table 1 lists the price of the local materials. Notably, the area is characterized by numerous rivers, particularly within a 25–30 km radius of the site, where several pebble quarries provide stable pebble sources. Given the engineering requirements and the principle of local sourcing, two local pebbles were chosen as the coarse aggregate for the concrete.

## 3. Materials and Methods

### 3.1. Raw Materials

**Figure 2:**
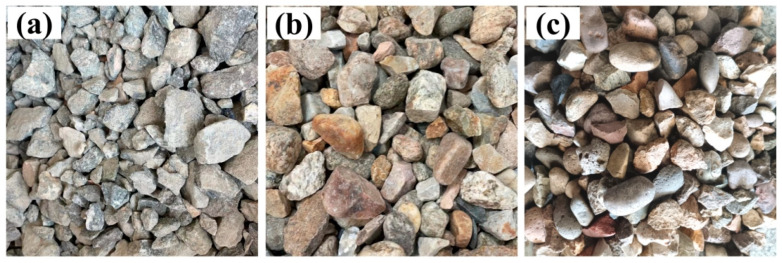
Physical appearance of the coarse aggregates: (**a**) crushed stone; (**b**) Pebble A; (**c**) Pebble B.

The raw materials used in this work were as follows:

(a) The cement used was Jinyu P·O 42.5, with a density of 3.11 g/cm^3^ and a specific surface area of 390 m^2^/kg. Additional physical properties are shown in Table 2, and its chemical composition is provided in Table 3.
(b) The fly ash used was class II, with a fineness (retained on a 45 μm square sieve) of 20.1%, a water demand ratio of 99%, a 7-day activity index of 95%, and a 28-day activity index of 78%. Its chemical composition is detailed in Table 3.
(c) Three types of coarse aggregates were used in the experiment. The crushed stone was supplied by a domestic construction material company, while the pebbles (Pebble A, and Pebble B) were sourced from local rivers in Bolivia. All three aggregates have a nominal maximum particle size of less than 26.5 mm. The appearance of these coarse aggregates is shown in Figure 2. The crushed stone aggregate exhibits an angular surface, while the pebbles display smoother surfaces and better sphericity than crushed stone.
(d) Fine Aggregate: the fine aggregate is Zone II, continuously graded, manufactured sand, with a fineness modulus of 2.5, a stone powder content of 7%, and a methylene blue value of 1.1.
(e) A polycarboxylate-based, high-performance, water-reducing agent was used, with a water reduction rate of 29% and a solid content of 15%.
(f) Tap water from the laboratory was used for the preparation of the concrete.

### 3.2. Concrete Preparation

Table 4 shows the concrete mix design used for this work. CS1 corresponds to the construction mix of the Sucre Highway Project. The mix design alters the type of coarse aggregate used or replaces 25% of the cement content with fly ash while keeping the amount of water-reducing agent constant.

During the mixing process, coarse aggregate, fine aggregate, cement, fly ash, and water were added sequentially to the mixing equipment. After achieving uniform mixing, tests were conducted on the fresh concrete. The fresh concrete was then poured into molds and left undisturbed for 2 days in the laboratory (at 50% relative humidity and 20 ± 5 °C) before demolding. Subsequently, the samples were transferred to a curing chamber (maintained at 95% relative humidity and 20 ± 2 °C) and cured until they were 28 days old. Finally, mechanical performance tests and permeability resistance tests were carried out on these samples.

### 3.3. Test Methods

According to JGJ 52-2006 [20], the crushing value, apparent density, and bulk density of the coarse aggregates were measured, and a sieve analysis was conducted.

The workability of the fresh concrete was tested in accordance with GB/T 50080-2016 [21]. This included the measurements of air content, slump, and slump flow.

According to JTG 3420-2020 [22], the specimens used to test splitting tensile strength and compressive strength were prisms with side lengths of 100 mm × 100 mm × 100 mm, while the specimen used to test the flexural tensile strength was the prism with side lengths of 100 mm × 100 mm × 400 mm. A microcomputer-controlled, electro-hydraulic servo universal testing machine (WEW-300E, BAIROU, China) was used to test the mechanical properties of hardened concrete with an age of 28 days. Splitting failure surfaces of the concrete were captured using a Digital Microscope (2 M 1920 × 1080, TOMLOV, China). The flexural tensile strength of the concrete was evaluated against the traffic load requirements specified in JTG D40-2011 [23].

The permeability resistance tests for concrete were conducted at 28 d age in accordance with GB/T 50082-2024 [24]. The permeability depth method evaluates the permeability of concrete by measuring the depth of liquid penetration within a specified time (24 h) and under specified pressure conditions (1.2 MPa). The specimen is a circular body with an inner diameter of 175 mm at the upper end and 185 mm at the lower end and a height of 150 mm. Using an automatic permeability tester (JYJC-146, RONG JI DA, China), the permeability resistance of concrete was expressed.

## 4. Results and Discussion

### 4.1. Properties of Coarse Aggregates

**Figure 3:**
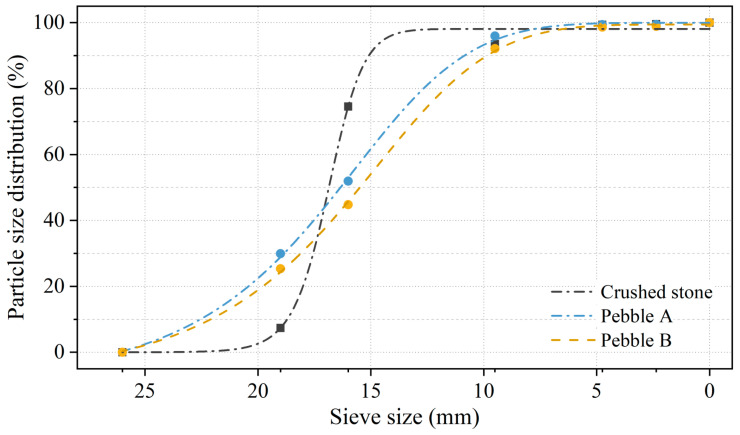
Grading curve for coarse aggregate.

To identify the technical characteristics of these different coarse aggregates, basic performance tests were conducted. As shown in Table 5, the crushed stone exhibited a density of 2820 kg/m^3^ and a crushing value of 4.9%. In comparison, the densities of the two pebbles were similar to each other but lower than that of the crushed stone, at approximately 2710 kg/m^3^. The crushing values of the pebbles were significantly higher, with Pebble B reaching 10.5%, nearly double that of the crushed stone. The grading curves of the coarse aggregates are presented in Figure 3. The grading curve for the crushed stone is steep, with the particle size distribution of the crushed stone concentrated within the 10–20 mm range. In contrast, the grading curves for the two pebbles are nearly identical and gradual, indicating a dispersed grading compared to the crushed stone. Notably, pebbles contain over 10% more 20–25 mm particles than crushed stone, consistent with their appearance in Figure 2.

The performance differences between the crushed stone and pebbles can be attributed to the differences in their parent rock materials. Crushed stone from quarries, primarily composed of igneous and metamorphic rocks, typically exhibits a higher strength and lower porosity. It exhibits a low-pressure crushing value of 4.9% and a high density of 2820 kg/m^3^, resulting in stability under load. On the other hand, pebbles are mainly composed of sedimentary rocks. Due to prolonged water erosion, their surfaces are smoother, which may provide better wear resistance, but generally leads to inferior mechanical properties compared to crushed stone. In addition, the difference in the gradation of different aggregates is related to their crushing process.

### 4.2. Workability of Fresh Concrete

The workability of fresh concrete was analyzed in terms of slump, slump flow, and air content. As shown in Table 6, CS1 exhibited an air content of 6.8% and a slump of 125 mm, but there was almost no change in the slump flow after removing the slump cone. When the crushed stone in CS1 was replaced with pebbles, the slump in PA1 and PA2 increased by 15 mm; however, the slump flow remained unchanged, and the air content increased by 0.4% to 0.5%. After the incorporation of fly ash, the workability of the concrete significantly improved. For the concrete prepared with the same type of aggregate, the air content increased by 1% compared to the groups without fly ash, while the slump increased by approximately 70 mm. Among these samples, the concrete prepared with Pebble B exhibited the highest slump flow and air content.

Typically, road construction projects impose lower workability requirements for pavement concrete. The concrete prepared based on the construction mix ratio (CS1, PA1, and PB1) exhibited a slump lower than 140 mm. Additionally, the high cement content resulted in a strong adhesive force within the paste, making it difficult to observe significant changes in flowability. However, due to the smooth surface of the pebbles, the frictional resistance of the concrete was reduced, which also led to an air content in the mixture of more than 7.2% [25,26]. However, the slump flow of the pebble concrete was constrained by the high cement content, showing little change. Since highway pavements are typically compacted using rollers, the workability of CS1, PA1, and PB1 still met the requirements of the construction mix design.

After the addition of fly ash, the fresh performance improved due to its filling effect and “ball-bearing” effect. Overall, the mixtures prepared with Pebble A and Pebble B demonstrated better workability, with a higher slump and slump flow. In particular, the mixture containing Pebble B exhibited the best flowability due to its smoother surface.

### 4.3. Mechanical Properties of Harden Concrete

#### 4.3.1. Splitting Tensile Strength

The mechanical properties and morphological characteristics of aggregates significantly influence the bond strength at the paste–aggregate interface, subsequently affecting the mechanical behavior of the concrete. The splitting tensile strength test mainly reflects the cracking resistance of concrete under lateral tensile stress, which is closely related to the strength of the bond between the aggregate and the cement paste. To analyze the impact of aggregate type on concrete performance, splitting tensile strength tests were conducted.

**Figure 4:**
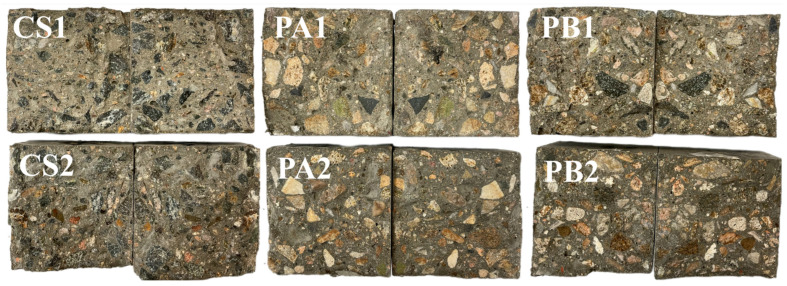
Split sections of the crushed stone concretes (CS1/CS2) and two kinds of pebble concrete (PA1/PA2 and PB1/PB2).

Figure 4 shows the splitting failure surfaces of the specimens. It can be observed that the internal color of the specimens containing fly ash (CS2, PA2 and PB2) is darker after symmetric splitting. From the fracture surfaces, the crushed stone aggregates appear irregular in shape, with sharp edges. The distribution of crushed stone within the concrete cross-section is uniform and dense, with strong bonding between the paste and the aggregates. The pebble aggregates, on the other hand, exhibit elliptical shapes with large coarse particles. Pebble B, in particular, demonstrates a higher degree of roundness.

**Figure 5:**
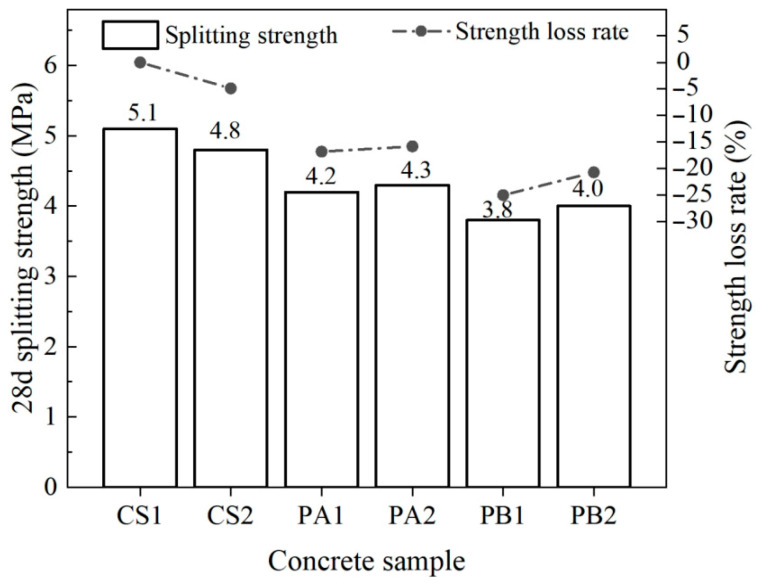
Splitting tensile strength variations in the concrete.

Figure 5 presents the splitting tensile strength results for each specimen. Concrete prepared with crushed stone, without fly ash, was used as a reference (CS1). CS1 exhibited the highest splitting tensile strength of 5.1 MPa. Changing the aggregate type significantly influenced the splitting tensile strength, with the concrete prepared using Pebble A and Pebble B showing strength reductions of 16.8% and 25.0%, respectively. In comparison, the incorporation of fly ash had a minor effect on the 28-day splitting tensile strength of concrete made with the same pebble aggregates, resulting in differences of less than 0.3 MPa. This indicates that the impact of fly ash on splitting tensile strength is relatively small.

**Figure 6:**
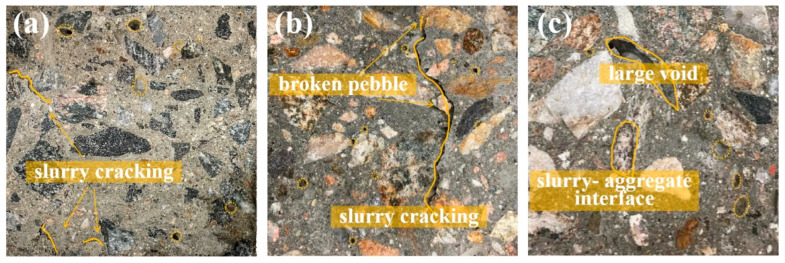
The splitting surface of concrete: (**a**) crushed stone concrete with slurry cracking; (**b**) pebble concrete with slurry cracking and aggregate fracture; (**c**) pebble concrete with interface debonding and large holes (The dotted yellow lines display the pores inside the concrete).

Bond strength is one of the critical factors determining the overall mechanical performance of concrete, as it dictates the rate and extent of crack propagation within the material under external forces [27]. Crushed stone, derived from quarried bedrock, presents a distinct angularity on its surface, as shown in Figure 6a. This angularity promotes strong mechanical interlocking with the cement paste, resulting in fewer defects at the splitting interface, which are primarily characterized by paste cracking. Since the crushing value of crushed stone is as low as 4.9%, the cracking of the concrete can be attributed to the failure of the paste. In contrast, Figure 6b reveals that the failure surface of pebble concrete shows a combination of cracked pebbles (Pebble A), paste fractures, and air voids concentrated around the aggregates. Pebble B, with a crushing value of up to 10.5%, exhibits both high surface smoothness and a relatively loose internal structure. As illustrated in Figure 6c, the embedding effect of Pebble B is relatively weak, and holes are present at the paste–aggregate interface, leading to large hollow areas within the concrete. The synergistic effect of the smooth surface and the weaker mechanical properties of the parent rock significantly contribute to the reduction in splitting tensile strength.

Furthermore, the addition of fly ash does not have a pronounced impact on splitting tensile strength. This can be attributed to the fact that the microstructural improvements introduced by fly ash compensate for the deterioration in splitting performance caused by the type of aggregate used. Therefore, pebble concrete experiences slight improvements in splitting performance. Overall, the type of aggregate remains the dominant factor affecting the splitting tensile strength of the concrete.

#### 4.3.2. Compressive Strength

**Figure 7:**
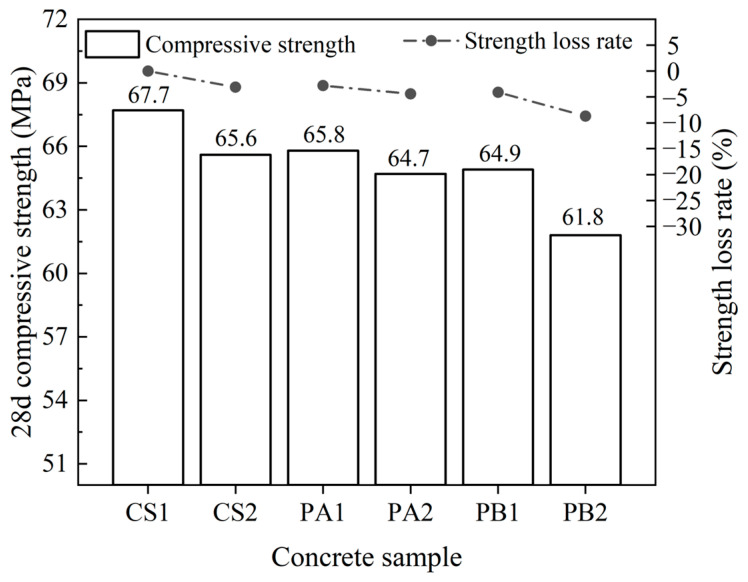
Compressive strength variations in the concrete.

Highway pavements must withstand repeated vehicular loads and various environmental factors, with the compressive strength of concrete being a key determinant of its long-term stability and load-bearing capacity. Figure 7 illustrates the compressive strength results for the tested concrete specimens, with crushed stone serving as the baseline. CS1 exhibited the highest compressive strength of 67.7 MPa. The compressive strength of concrete decreases progressively when prepared with crushed stone, Pebble A, and Pebble B as coarse aggregates, but the reduction range was less than 6 MPa. The strength loss remained within 5%, which is considered acceptable. Upon the incorporation of 25% fly ash, the trend of a compressive strength reduction in concrete prepared with pebbles as a coarse aggregate followed a similar pattern to that observed without fly ash. However, in the presence of both Pebble B and fly ash, the compressive strength of B2 showed a significant reduction, with a decrease of 8.7%.

The type of aggregate significantly impacts the compressive strength of concrete. Crushed stone, with a crushing value of 4.9%, provides substantial mechanical support within the concrete matrix. While the introduction of fly ash reduces the amount of cement by 25%, thereby slightly decreasing compressive strength by 1–3 MPa, the fly ash likely contributes a filling effect and a potential pozzolanic reaction, resulting in an acceptable strength loss.

Pebble A, with a crushing value of 8.3%, has a looser internal structure compared to crushed stone. Thus, the compressive strength of concrete prepared with Pebble A is about 2 MPa lower than that of crushed stone concrete. After incorporating fly ash, the strength loss rate of PA2 is 4.1%, indicating that the filling and pore structure improvement effects of fly ash are limited. Pebble B, with the highest crushing value of 10.5%, provides the weakest structural support in the concrete. The addition of fly ash leads to a further decrease in compressive strength, with the strength loss rate reaching 8.7%, resulting in a significant reduction in compressive strength. Overall, crushed stone concrete exhibits the highest compressive strength, followed by concrete prepared with Pebble A, while concrete prepared with Pebble B shows the lowest compressive strength and the highest strength loss rate, corresponding to the crushing performance of the aggregates used in the concrete.

#### 4.3.3. Flexural Tensile Strength

**Figure 8:**
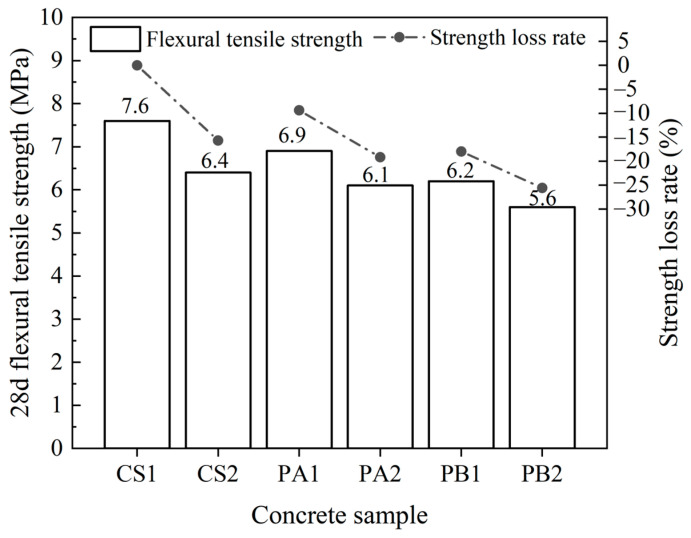
Flexural tensile strength variations in the concrete.

In highway pavements, the concrete must not only withstand vertical pressures but also resist bending stresses caused by the uneven distribution of vehicle loads. Flexural tensile strength is a critical indicator of pavement resistance to bending failure. As shown in Figure 8, the variation in flexural tensile strength follows a similar trend to that of compressive strength. According to the JTG D40-2011 [23], all concrete specimens met the flexural tensile strength requirements for extremely heavy, very heavy, and heavy traffic loads (≥5.0 MPa). CS1 demonstrated the highest flexural tensile strength of 7.6 MPa, while the use of Pebble A and Pebble B as aggregates led to reductions of approximately 9.4% and 18.0%, respectively, in flexural tensile strength compared to concrete prepared with crushed stone. These strength losses are significant. The addition of 25% fly ash further exacerbates the decline, with flexural tensile strength decreasing by an additional 9.8% and 7.6% for concrete containing Pebble A and Pebble B, respectively, compared to their non-fly ash counterparts. Both the aggregate type and the incorporation of fly ash significantly impact flexural tensile strength.

Aggregates function as the structural framework within the concrete, influencing its mechanical properties in a manner similar to that observed for compressive strength. The use of crushed stone, Pebble A, and Pebble B in sequence resulted in a decrease in flexural tensile strength. However, flexural tensile strength is also closely related to the bond quality at the paste–aggregate interface. The smoother surface of pebbles compared to crushed stone weakens this bond, leading to a reduction in flexural tensile strength. Crushed stone, with its high apparent density of 2820 kg/m^3^ and low crushing value of 4.9%, provides stronger mechanical support. Its angular shape also promotes tighter bonding with the paste, which enhances its ability to resist bending stresses.

The flexural tensile strength of PA1 and PB1 was reduced by approximately 9.4% and 18.02%, respectively, compared to CS1. This decrease is primarily attributed to the higher crushing values and smoother surfaces of pebbles, which make the concrete more prone to micro-cracks during bending stress. These cracks may develop due to either aggregate fracture or debonding at the paste–aggregate interface, reducing the concrete’s ability to resist bending. The incorporation of fly ash likely introduces additional micro-voids, further weakening the flexural tensile strength of the concrete. The flexural tensile strength of PB1 was approximately 8.6% lower than that of PA1. As Pebble B has the lowest crushing value of 10.5%, PB1 showed the poorest flexural performance among the tested concretes. The addition of fly ash, by increasing the porosity, further compromises the flexural tensile strength. The internal structure easily breaks under bending stress, which exerts a pronounced negative effect on the overall flexural performance.

### 4.4. Impermeability of Harden Concrete

Pavement concrete is directly exposed to rain, snow, and chemical environments, making impermeability a critical factor affecting its long-term durability. For concrete prepared with crushed stone, the seepage height for both CS1 and CS2, with or without fly ash, remained consistently low at 6 mm. Meanwhile, the seepage height of PA1 and PB1 reached 7 mm and 9 mm, respectively. However, after the addition of fly ash, the type of aggregate no longer influenced the permeability performance. The seepage heights of PA2 and PB2 dropped back to 6 mm, aligning with the performance of the crushed stone concrete.

The rough surface texture of crushed stone enhances the mechanical interlocking between the aggregate and the cement paste, resulting in a stronger bond at the paste–aggregate interface. This reduces the likelihood of water permeating through the interface. In crushed stone concrete, although fly ash can change the microstructure, the significant volume of the aggregate means that the interface improvement brought by crushed stone plays a dominant role in the impermeability of the concrete. Additionally, crushed stone concrete exhibited the lowest air content of 6.8%, which reduces the number of voids and further prevents water infiltration. As a result of these combined factors, both CS1 and CS2 exhibited a low water penetration depth of 6 mm.

When pebbles replace crushed stone as the coarse aggregate, the smoother surface of the pebbles, particularly Pebble B, leads to a weaker bond at the paste–aggregate interface and the formation of micro-voids. This increases the potential for water to penetrate along the paste–aggregate interface, leading to a higher water penetration depth of 9 mm. However, unlike crushed stone concrete, the addition of fly ash to pebble concrete plays a dominant role in improving the impermeability through its effect on the microstructure. The decline in impermeability caused by the use of pebbles is effectively compensated by the fly ash, resulting in a significant improvement in impermeability for pebble concrete, which ultimately achieved the same performance level as crushed stone concrete.

## 5. Conclusions

This study was conducted based on the specific requirements of the Sucre Highway project, focusing on the use of local pebbles as coarse aggregates. The major conclusions are summarized as follows:

1. Crushed stone aggregate exhibited a crushing value of 4.9%, representing the highest compressive strength. Pebble A and Pebble B had crushing values of 8.3% and 10.5%, respectively. Despite their similar physical properties, both pebbles were inferior to crushed stone in terms of performance, and their gradation was dispersed.
2. The type of aggregate and the addition of fly ash significantly influenced the fresh properties of concrete. Due to the smoother surface of pebbles, air entrapment in concrete increased, resulting in higher air content and greater slump, although the impact on flowability was minimal. In contrast, the incorporation of fly ash led to pronounced changes in fresh concrete performance, including a 1% increase in air content, a 70 mm increase in slump, and noticeable improvements in flowability.
3. The primary failure modes of pebble concrete included matrix cracking, aggregate fracture, and interface debonding. The type of aggregate and the presence of fly ash had only a minor effect on compressive strength. All concrete types met the flexural tensile strength requirements for heavy/very heavy/extra heavy traffic load levels. However, aggregate crushing value, surface characteristics, and the inclusion of fly ash had significant impacts on flexural tensile strength. Compared to crushed stone, flexural tensile strength decreased by 9.4% and 18.0% for Pebble A and Pebble B concrete, respectively. The addition of fly ash resulted in strength losses, with reductions of 15.7%, 19.2%, and 25.6%, respectively, for the three types of coarse aggregates.
4. In crushed stone concrete, the improvement in the interface due to the inclusion of the crushed stone was the dominant factor influencing impermeability, resulting in a water penetration depth of 6 mm. In pebble concrete, however, the improvement in microstructure brought about by fly ash became the primary factor affecting impermeability, enabling the impermeability performance to reach the same level as that of crushed stone concrete. The addition of 25% fly ash achieves a synergy between the utilization of local pebble resources and the durability guarantee of concrete.

In summary, from the perspectives of resource conservation and engineering quality, the comprehensive performance analysis suggests the use of Pebble A as the coarse aggregate, combined with the addition of 25% fly ash, to optimize the concrete mix for pavement. This approach achieves a green concrete formulation for the highway project while ensuring construction quality.

## Figures and Tables

**Table 1:** Prices of materials.

Local Material	Unit Price/(BS/t)	Description
Cement	750	Freight included
Sand	40	Excluding freight
Local crushed stone	80	Excluding freight
Pebble A	40	Excluding freight
Pebble B	40	Excluding freight

**Table 2:** Physical properties of the cement.

Setting Time/Min		Compressive Strength/MPa		Flexural Tensile Strength/MPa	
Initial	Final	3 d	28 d	3 d	28 d
190	300	30.90	59.30	5.20	8.60

**Table 3:** Physical properties of the cement (%).

Binder	SiO_2_	Al_2_O_3_	CaO	MgO	K_2_O	Na_2_O
Cement	21.96	4.19	65.98	2.25	0.59	0.27
Fly ash	65.23	20.98	3.09	2.02	2.12	1.37

**Table 4:** Mixing ratio (kg/m^3^).

Sample	Water	Binder		Coarse Aggregate			Sand	Water Reducing Agent
		Cement	Fly Ash	Crushed Stone	Pebble A	Pebble B		
CS1	170	420	/	1018	/	/	700	10.8
CS2	170	315	105	1018	/	/	700	10.8
PA1	170	420	/	/	1018	/	700	10.8
PA2	170	315	105	/	1018	/	700	10.8
PB1	170	420	/	/	/	1018	700	10.8
PB2	170	315	105	/	/	1018	700	10.8

**Table 5:** Physical properties of coarse aggregates.

Coarse Aggregate Type	Crushing Value (%)	Apparent Density (kg/m^3^)	Bulk Density (kg/m^3^)	Void Content (%)
Crushed stone	4.9	2820	1452	48.5
Pebble A	8.3	2708	1430	47.2
Pebble B	10.5	2712	1431	47.2

**Table 6:** Workability of the fresh concrete.

Sample	Air Content (%)	Slump (mm)	Slump Flow (mm)	Sample	Air Content (%)	Slump (mm)	Slump Flow (mm)
CS1	6.8	125	/	CS2	7.8	190	510
PA1	7.2	140	/	PA2	8.1	210	530
PB1	7.3	140	/	PB2	8.3	205	565

## Data Availability

The raw data supporting the conclusions of this article will be made available by the authors on request.
